# Cancer-Associated Fibroblasts in the Hypoxic Tumor Microenvironment

**DOI:** 10.3390/cancers14143321

**Published:** 2022-07-07

**Authors:** Iljin Kim, Sanga Choi, Seongkyeong Yoo, Mingyu Lee, In-San Kim

**Affiliations:** 1 Department of Pharmacology and Research Center for Controlling Intercellular Communication, Inha University College of Medicine, Incheon 22212, Korea; choisa@inha.edu (S.C.); yoosk9501@inha.edu (S.Y.); 2 Division of Allergy and Clinical Immunology, Department of Medicine, Brigham and Women’s Hospital, Harvard Medical School, Boston, MA 02115, USA; mlee96@bwh.harvard.edu; 3 KU-KIST Graduate School of Converging Science and Technology, Korea University, Seoul 02841, Korea; 4 Medicinal Materials Research Center, Biomedical Research Institute, Korea Institute Science and Technology, Seoul 02792, Korea

**Keywords:** cancer, cancer-associated fibroblast, hypoxia, tumor microenvironment

## Abstract

**Simple Summary:**

Cancers have regions of low oxygen concentration where hypoxia-related signaling pathways are activated. The hypoxic tumor microenvironment has been widely accepted as a hallmark of cancer and shown to be a critical factor in the crosstalk between cancer and stromal cells. Fibroblasts are one of the most abundant cellular components in the tumor stroma and are also significantly affected by oxygen deprivation. In this case, we discuss the molecular and cellular mechanisms that regulate fibroblasts under hypoxic conditions and their effect on cancer development and progression. Unraveling these regulatory mechanisms could be exploited in developing potential fibroblast-specific therapeutics for cancer.

**Abstract:**

Solid cancers are composed of malignant cells and their surrounding matrix components. Hypoxia plays a critical role in shaping the tumor microenvironment that contributes to cancer progression and treatment failure. Cancer-associated fibroblasts (CAFs) are one of the most prominent components of the tumor microenvironment. CAFs are highly sensitive to hypoxia and participates in the crosstalk with cancer cells. Hypoxic CAFs modulate several mechanisms that induce cancer malignancy, such as extracellular matrix (ECM) remodeling, immune evasion, metabolic reprogramming, angiogenesis, metastasis, and drug resistance. Key signaling molecules regulating CAFs in hypoxia include transforming growth factor (TGF-β) and hypoxia-inducible factors (HIFs). In this article, we summarize the mechanisms underlying the hypoxic regulation of CAFs and how hypoxic CAFs affect cancer development and progression. We also discuss the potential therapeutic strategies focused on targeting CAFs in the hypoxic tumor microenvironment.

## 1. Introduction

### 1.1. Normal Fibroblasts and CAFs

Fibroblasts are one of the most abundant cells in the connective tissue, producing various ECM proteins. Fibrous structural proteins, adhesive proteins, and gelatinous ground substances secreted by fibroblasts form the three-dimensional framework of tissues. Fibroblasts are functionally heterogeneous in different organs, and even within the same organ, their roles may differ depending on their location. Heterogeneous phenotypes of fibroblasts can appear through different transcriptional programs depending on the epigenetic modifications and local environment [1,2,3]. Most fibroblasts originate from the primitive mesenchyme and are defined by their morphology and location within the resident tissue. Since fibroblasts share mesenchymal lineages with adipocytes, osteoblasts, and chondrocytes, they lack specific intrinsic markers and are distinguished by cell shape and localization combined with excluding the expression of markers assigned to other cell types [4,5,6]. Functionally, in addition to producing ECM proteins, fibroblasts can secrete a variety of signaling factors and metabolites that can affect surrounding cells. In response to tissue damage, they can temporarily transform into a highly contractile phenotype called myofibroblasts. This fibroblast subtype is commonly associated with α-smooth muscle actin (α-SMA) and are primarily involved in wound healing and tissue fibrosis [7,8,9]. Fibroblasts also participate in immune cell recruitment and contribute to regulating active or inhibitory responses in inflammatory conditions [10,11,12]. Therefore, the role of fibroblasts is not limited to ECM regulation but is also important for maintaining tissue homeostasis and communicating with other cells.

Normal fibroblasts usually exert inhibitory functions against cancer, whereas fibroblasts affected by cancer cells can be converted into CAFs, leading to a variety of cancer-promoting events. However, recent studies have shown that the relationship between cancer cells and fibroblasts is complex and context-dependent, with consequences that may be either positive or negative for cancer progression [13,14]. For instance, fibroblast-specific protein 1 (FSP1)-positive fibroblasts have been shown to prevent epithelial malignancies by protecting epithelial cells from DNA damage through collagen encapsulation of carcinogens [15]. CAFs can be roughly defined if they show an elongated morphology, lack the mutations found in cancer cells, and are negative for markers expressed in other cell types. CAFs are highly heterogeneous, and several cell surface markers have been used to define CAF subtypes, such as α-SMA, fibroblast activation protein (FAP), SP1, platelet-derived growth factor receptor (PDGFR), and tumor endothelial marker 8 (TEM8). However, none of these markers are exclusively expressed by CAFs, which is one of the factors that makes CAF-targeted therapy difficult [7,16]. Most CAFs originate from fibroblasts residing in cancer tissues and can be activated by multiple factors. Signaling molecules that induce CAF activation include TGF, interleukin-1 (IL-1), IL-6, tumor necrosis factor (TNF), PDGF, and fibroblast growth factor (FGF) [17,18,19,20,21]. Direct contact of fibroblasts with cancer cells can activate fibroblasts through membrane proteins such as Notch and Ephrin receptors [22,23,24,25]. Physiological damage caused by radiation, reactive oxygen species (ROS), or anticancer drugs can also activate CAF. Changes in the ECM environment, such as increased tissue stiffness, induce CAF through activation of various transcriptional programs [4]. Activated CAF stimulates tumor cells to form a positive feedback loop and reorganizes the tumor microenvironment to be more favorable for cancer growth. CAFs secrete proteases to break matrix crosslinking and reconstruct tumor tissue through various matrix components and crosslinking enzymes. Tissue reconstruction by CAF increases the stiffness of the tumor tissue and creates a pathway for cancer cells to invade more easily [2,4,6,20]. Increased tissue stiffness also causes blood vessels to collapse, leading to tumor hypoxia. This stimulates the survival and proliferation signals of cancer cells and reduces drug delivery [26].

### 1.2. Hypoxia Signaling Pathways

**Figure 1:**
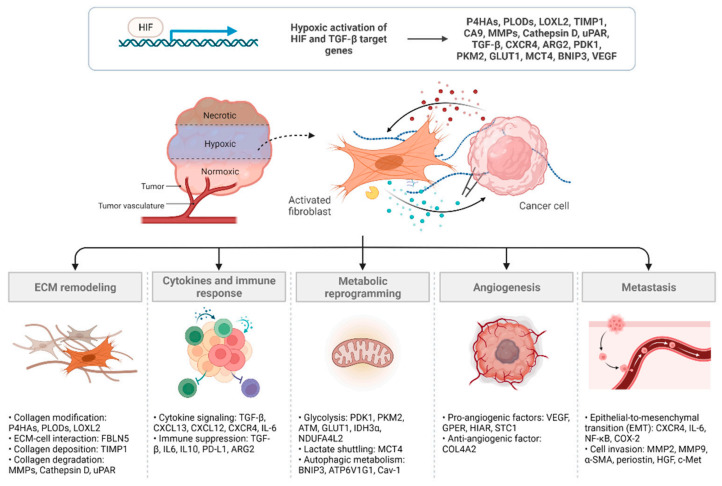
CAF-mediated cancer progression in hypoxia. Several mechanisms are involved in CAF-mediated cancer progression under hypoxia. HIF and TGF-β pathways play a major role in CAF activation and function. A number of genes have been demonstrated as direct transcriptional targets of HIF in either CAFs or cancer cells. Crosstalk between CAFs and cancer cells may alter ECM structure, immune responses, cell metabolism, angiogenesis, and metastasis through various signaling molecules (created with BioRender.com on June 2022).

Cancer has regions of permanent or temporary exposure to hypoxia due to abnormal blood vessel formation and lack of blood supply. As cancer grows, oxygen becomes more deprived, and hypoxia signaling is activated in both cancer cells and stromal cells in the tumor microenvironment. HIF transcription factors are thought to be the most important molecules of hypoxia signaling in cancer cells [27]. HIF-dependent signaling promotes adaptation of cells to hypoxic conditions, promoting changes favorable to cancer progression. HIF consists of a cytoplasmic α subunit that is degraded in the presence of oxygen and a constitutively expressed nuclear β subunit (also known as aryl hydrocarbon receptor nuclear translocator, ARNT) [28]. The HIF heterodimer interacts with CBP/p300 coactivator in the nucleus to transactivate downstream genes which contain hypoxia-responsive elements (HREs) [29]. The α subunit has two proline residues, which are hydroxylated by HIF prolyl hydroxylase (PHD; also known as Egl nine homolog, EGLN) enzymes in the presence of oxygen. Hydroxylation of proline residues promotes binding of HIF-α to von Hippel-Lindau tumor suppressor (VHL) and consequently ubiquitinates and degrades HIF-α. In the absence of oxygen, HIF-α is stabilized and translocate to the nucleus, where it binds to the β unit and activates the expression of hypoxia-related genes [30,31,32,33]. On the other hand, factor inhibiting HIF (FIH) inhibits the binding of HIF to the nuclear coactivators CBP/p300 by hydroxylating an asparagine residue in the C-terminal transactivation domain of HIF, thereby reducing transcriptional activity. In hypoxia, FIH enzyme activity is inhibited, and CBP/p300 binds to HIF, increasing transcriptional activity [34,35,36]. The HIF protein family consists of three members: HIF-1, HIF-2, and HIF-3. Although HIF-1 and HIF-2 are highly conserved at the protein level and share similar domain structures, their expression levels in specific tissues and the target genes they activate are quite different. There are several hundred known HIF-1/2 targets involved in cellular adaptation to hypoxia. HIF-2 is thought to be responsible for the long-term hypoxic response when HIF-1α is degraded after an acute hypoxic response. In contrast, HIF-3 lacks the transactivation domain found in HIF-1/2 and rather encodes a polypeptide that represses HRE-responsive gene expression [37]. Hypoxia promotes HIF-induced transcriptional responses in cancer cells as well as noncancerous stromal cells, including CAFs. CAFs are prominent component of the tumor microenvironment and are regulated under hypoxia by both HIF dependent and independent mechanisms [26,38]. In this review, we discuss the mechanisms by which hypoxia regulates CAFs and the role of activated CAFs in the hypoxic tumor microenvironment (Figure 1).

## 2. Mechanisms Underlying CAF Regulation and Function in Hypoxia

### 2.1. ECM Remodeling

Hypoxia and HIF have been implicated in the regulation of post-translational modification of collagen proteins and interaction between ECM components. Collagen prolyl hydroxylases (P4HAs) and lysyl hydroxylases (PLODs) are key enzymes involved in collagen deposition and fiber alignment. P4HA-mediated proline hydroxylation induces proper folding of newly synthesized procollagen chains and stabilizes the protein by increasing the melting temperature of collagen. PLODs hydroxylate the lysyl residues of collagen and form a cross-link between collagen and pyridinoline, which is necessary for collagen stabilization [39,40,41,42]. It has been shown that HIF transcriptionally activates both P4HAs and PLODs in fibroblasts to regulate collagen biogenesis and deposition. Fibroblast-specific HIF activation promotes ECM alignment and stiffness, which contributes to morphological changes and migratory behavior of breast cancer cells [43]. In lung fibroblasts, oxidative stress induces HIF activation by inhibiting FIH, which negatively regulates HIF, and increases the expression of PLOD2 and lysyl oxidase-like 2 (LOXL2). Lysyl oxidases are essential enzymes in the biosynthesis of connective tissue that catalyze the formation of cross-links in collagen and elastin. FIH inhibition by siRNA-mediated knockdown or hydroxylase inhibitors increased collagen cross-linking and tissue stiffness [44]. In pancreatic ductal adenocarcinoma, hypoxia upregulates fibulin-5 (FBLN5) expression via TGF-β and phosphoinositide 3-kinase (PI3K)/protein kinase B (AKT) signaling. FBLN5 is a pro-tumorigenic matricellular glycoprotein that inhibits fibronectin-integrin binding required for ECM-cell interaction. Hypoxic induction of FBLN5 in CAFs isolated from mouse pancreatic ductal adenocarcinoma were reversed by pharmacologic inhibition of TGF-β or PI3K/AKT [45].

Hypoxia induces collagen expression and secretion. In renal fibroblasts, via HIF-1α, hypoxia induces the production of collagen proteins and decreases the turnover of ECM structure. HIF-1α transcriptionally activates TIMP metallopeptidase inhibitor 1 (TIMP1) to suppress matrix metalloproteinases (MMPs) and ECM turnover [46]. It has been suggested that renal pericytes can be transformed into myofibroblasts by hypoxia, possibly via VEGF and PDGF signaling, to enhance ECM production and fibrosis [47]. Likewise, type I and III collagens were upregulated by hypoxia in rat cardiac fibroblasts [48]. In keloid tumors, HIF-1α protein and target genes were increased in keloid fibroblasts compared to normal dermal fibroblasts. Indeed, hypoxic stress stimulated CAF-mediated collagen secretion, which was inhibited by CAY10585, a selective HIF-1α inhibitor [49]. Hypoxic areas within the tumor mass change over time into fibrous foci replacing necrotic lesions. In human breast carcinoma, immunohistochemical studies have shown that fibrosis localized to hypoxic regions correlates with the expression of HIF-1 target gene carbonic anhydrase 9 (CA9) [50]. CA9 is a transmembrane glycoprotein with an enzymatic activity that extrudes acid into the extracellular space and stabilizes the intracellular pH, thereby preventing acidosis-induced apoptosis of cells [51]. Indeed, it has been shown that ectopic expression of CA9 in NIH3T3 fibroblasts promotes their proliferation [52]. CA9 is highly expressed in hypoxic CAFs and to a lesser extent in tumor cells and is associated with higher recurrence and poor survival rates in breast cancer patients [50]. However, further in vitro and in vivo studies are needed to elucidate the mechanism of collagen biogenesis in hypoxic CAFs and cancer cells.

**Figure 2:**
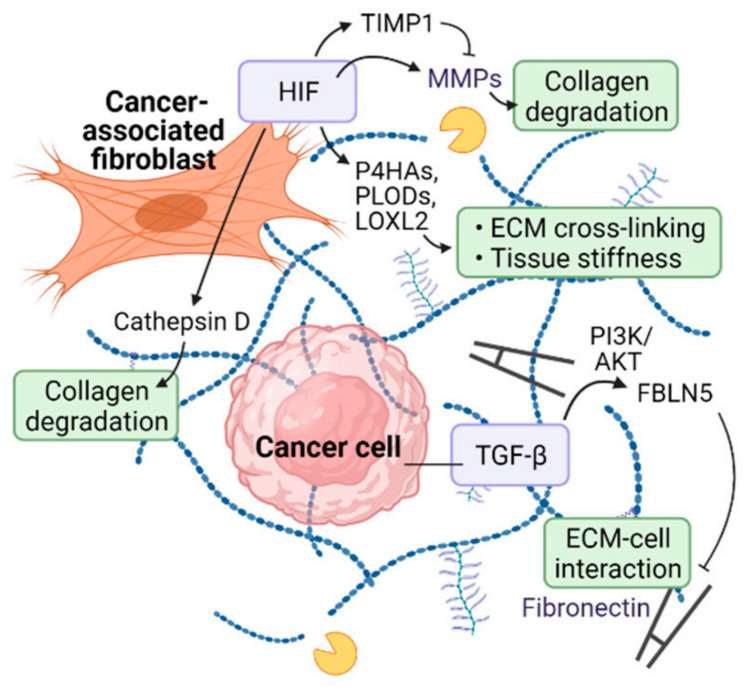
CAF-mediated ECM remodeling in hypoxia. Hypoxia regulates extracellular environment via several mechanisms: post-translational modification of ECM proteins; ECM cross-linking; increasing ECM stiffness; collagen synthesis; collagen degradation; altering ECM-cell interactions. Abbreviations: HIF, hypoxia-inducible factor; TIMP1, TIMP metallopeptidase inhibitor 1; MMP, matrix metallopeptidase; P4HA, collagen prolyl hydroxylase; PLOD, lysyl hydroxylases; LOXL2, lysyl oxidase-like 2; TGF-β, transforming growth factor beta; PI3K, phosphoinositide 3-kinase; AKT, AKT serine/threonine kinase; FBLN5, fibulin (created with BioRender.com on June 2022).

In addition, ECM remodeling is also attributed to the degradation of collagen proteins. Hypoxia-induced degradation of ECM structure may contribute to tumor cell invasion and metastasis by creating a physical route for intra- and extravasation. MMP family of proteinases specifically target various ECM components, such as collagens and gelatins, for proteolytic degradation. HIF transcription factors have been shown to upregulate the expression of several MMP proteins including MMP1, MMP2, MMP9, and MMP14 under hypoxia [53,54,55,56]. Aspartyl proteinase cathepsin D is another matrix degrading enzyme induced by hypoxia or HIF-1α overexpression [54]. Cathepsin D is aberrantly expressed in breast cancer patients and contributes to the invasion of metastatic cancer cells via collagen degradation [57,58,59]. Urokinase plasminogen activator receptor (uPAR), transcriptionally activated by HIF-1α, increases the metastatic potential of cancer cells under hypoxic conditions [54,60,61]. HIF-1-driven CA9 expression acidifies the extracellular environment, which in turn activates CAFs to produce MMP2 and MMP9 for ECM breakdown and tumor cell invasion in prostate cancer [62]. In summary, hypoxic signaling engages in the production, modification, and degradation of matrix proteins that contribute to ECM remodeling within the tumor microenvironment (Figure 2).

### 2.2. Cytokines and Immune Response

In early studies, hypoxia has been shown to regulate several hypoxia-related genes in human fibroblasts [63]. Representative genes include well-known HIF targets such as TGF-β. It has been reported that hypoxia induces the expression of fibrogenic cytokine TGF-β, which is involved in matrix protein production and transition of fibroblasts to myofibroblasts [64,65]. Myofibroblasts are a fibroblast subpopulation that can be transformed from resident fibroblasts in the tumor microenvironment by several factors, including hypoxia. Myofibroblasts are characterized by the expression of α-SMA within cytoplasmic stress fibers, and sustained activation of myofibroblasts can lead to tissue fibrosis. The stiff ECM environment generated by cancer-associated myofibroblasts increase the risk of epithelial–mesenchymal transition and invasiveness of tumor cells [66]. It has been shown that hypoxia activates TGF-β signaling via HIF-1α to convert fibroblasts into myofibroblasts, which in turn induces C-X-C motif chemokine 13 (CXCL13) expression for B cell recruitment and metastatic progression of prostate cancer cells. Nuclear translocation of HIF-1α was markedly increased in fibroblasts isolated from mouse prostate cancer model and deletion of HIF-1α prevented the hypoxic induction of fibroblast-to-myofibroblast differentiation [67]. CAFs also secrete CXCL12 (also known as stromal cell-derived factor 1, SDF1), which is a canonical ligand for the chemokine receptor CXCR4, to promote the growth and angiogenesis of CXCR4-expressing cancer cells [68]. CXCR4 has been identified as a direct transcriptional target of HIF-1α under hypoxia in various cell types including mouse embryonic fibroblasts (MEFs). In addition, hypoxia enhances CXCR4 mRNA stability, thereby regulating CXCR4 expression at both transcriptional and post-transcriptional levels [69]. In another study, CAF has been shown to induce HIF-1α-dependent oxidative response, thereby increasing the expression of CXCR4 and interleukin-6 (IL-6) receptors in prostate cancer cells to promote epithelial-to-mesenchymal transition (EMT) and invasion of the tumor cells [70].

CAFs are known to support cancer cells evade surveillance of the immune system by secreting various factors that can regulate tumor-associated immune cells [12,71]. In melanoma, CAFs were shown to secrete a number of immunomodulatory factors such as TGF-β, IL6, IL10, and PD-L1 to inhibit T cell-mediated cytotoxicity [72]. In pancreatic ductal carcinoma, there was an aberrant expression of the arginase 2 (ARG2) in hypoxic CAFs. ARG is an enzyme that metabolizes arginine, an important factor required for T cell survival and activation. Increased consumption of arginine by ARG2-overexpressing CAFs restricted T cell proliferation and anti-cancer immune reactions. ARG2 has a potential HRE sequence, and upregulation of ARG2 by hypoxia is associated with HIF-1α expression [73].

The paracrine actions of cytokines and growth factors secreted by CAFs also confer resistance to radiation therapy. CAF-derived factors such as IGF2, PDGF-AA, and insulin-like growth factor binding proteins (IGFBPs) were found in conditioned media that increased the protective effects, survival, and proliferation of HeLa cells against irradiation [74]. In addition, radiation therapy itself promotes CAF activation by producing ROS and inducing inflammation [75]. Radiotherapy induces spatial and temporal fluctuations in oxygen concentrations, which potentiates the fibrotic and pro-angiogenic responses and immune modulation within the tumor microenvironment [76,77]. Therapeutic attempts to restore oxygenation before or during radiation therapy may be helpful.

### 2.3. Metabolic Reprogramming

Altered metabolic function is observed in many types of cancers and aerobic glycolysis is considered as one of the major hallmarks of cancer. One study showed that fibroblasts transformed with constitutively active HIF-1α mutant promote the in vivo growth of co-injected MDA-MB-231 breast cancer cells via enhanced aerobic glycolysis and paracrine production of nutrients [78]. Similarly, HIF-1α upregulated the expression of glycolytic enzymes and lactate production in human synovial fibroblasts to support energy metabolism and cell survival [79]. In dermal fibroblasts cultured in lactate medium, HIF-1α was stabilized due to ROS production and the expression of glycolytic enzymes such as pyruvate dehydrogenase kinase 1 (PDK1) and pyruvate kinase M2 (PKM2) was increased [80].

As in cancer cells, the change in glucose metabolism is one of the characteristic features of CAFs. It has been shown that hypoxia enhances glycolysis in mammary CAFs through oxidized activation of the ATM serine/threonine kinase. ROS-activated ATM induces glucose transporter 1 (GLUT1) membrane translocation via phosphorylating GLUT1 at Ser-490. Membrane GLUT1 uptakes glucose from ECM for glycolysis and lactate production. In addition, ATM upregulates PKM2 expression possibly via the PI3K/AKT pathway to enhance glycolytic activity. Furthermore, lactate generated by hypoxic CAFs promotes breast cancer cell invasion by activating the TGF-β1/p38 MAPK pathway and upregulating MMP2 and MMP9 expressions [81]. In a following study, hypoxia-activated ATM phosphorylated BCL2 interacting protein 3 (BNIP3) to induce autophagy and exosome release in mammary CAFs. ATM also phosphorylates ATPase H^+^ transporting V1 subunit G1 (ATP6V1G1) to induce fusion between autophagosomes and multivesicular bodies. Hypoxic CAFs promote cancer cell invasion through this autophagy-related exosome release [82].

Chronic hypoxia reprograms normal fibroblasts into CAFs that promote glycolysis for breast cancer progression. Hypoxia stimulates glycolytic CAFs to provide lactate to cancer cells, promoting biosynthetic processes such as the pentose phosphate pathway (PPP) and nucleotide metabolism. Mechanistically, hypomethylation of HIF1A promoter in hypoxic CAFs led to sustained elevation of HIF-1α and pro-glycolytic HIF-1α target genes. This epigenetic modification maintains long-term and persistent transcriptome changes despite reoxygenation after hypoxic exposure [83]. In prostate cancer, direct intercellular contact with cancer cells triggers CAFs to undergo metabolic rewiring toward glycolytic metabolism. Sirtuin 3 (SIRT3)-dependent ROS production and HIF-1α stabilization upregulates the expression of glucose transporter GLUT1 and lactate transporter monocarboxylate transporter-4 (MCT4) in CAFs, thereby shuttling lactate from CAFs to cancer cells to promote anabolic processes and cell growth [84]. Moreover, hypoxia has been shown to enhance glycolysis and upregulate MCT4 expression via TGF-β1-induced autophagy in CAFs [85].

In tricarboxylic acid (TCA) cycle, isocitrate dehydrogenase (IDH) enzymes catalyze the decarboxylation of isocitrate to produce α-ketoglutarate (also known as 2-oxoglutarate) and carbon dioxide [86]. It has been shown that downregulation of IDH3α in CAFs switches the cancer cell metabolism to aerobic glycolysis by stabilizing HIF-1α, but not HIF-2/3α, in an α-ketoglutarate-dependent manner. In TGF-β- or PDGF-induced CAF models, aberrant expression of miR-424 inhibited IDH3α mRNA by targeting its three prime untranslated region (3′-UTR). Downregulation of IDH3α reduces the effective level of α-ketoglutarate, which in turn limits the activity of PHD2 to hydroxylate HIF-1α for degradation. Stabilized HIF-1α transactivates glycolytic enzymes and decreases oxidative phosphorylation via upregulating NADH dehydrogenase (ubiquinone) 1 alpha subcomplex, 4-like 2 (NDUFA4L2) protein [87].

**Figure 3:**
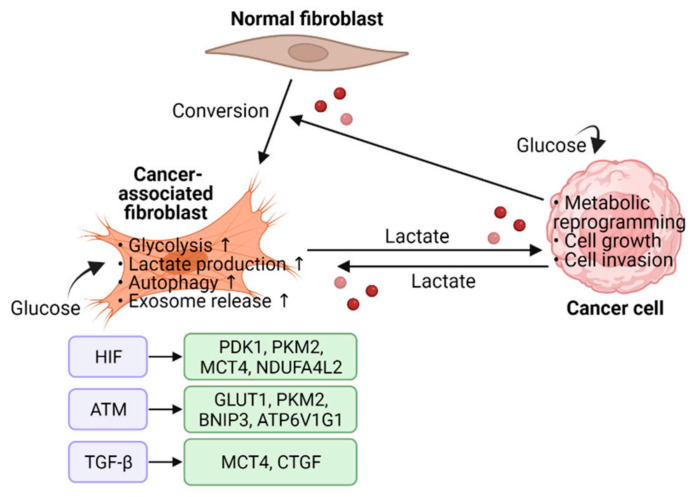
CAF-mediated metabolic reprogramming in hypoxia. Hypoxia regulates cell metabolism via several mechanisms: switching of glucose metabolism; increasing glucose uptake; lactate production; lactate shuttling; autophagic activation; exosome release. Abbreviations: HIF, hypoxia-inducible factor; PDK1, pyruvate dehydrogenase kinase 1; PKM2, pyruvate kinase M1/2; MCT4, monocarboxylic acid transporter 4; NDUFA4L2, NADH dehydrogenase (ubiquinone) 1 alpha subcomplex, 4-like 2; GLUT1, glucose transporter 1; BNIP3, BCL2 interacting protein 3; ATP6V1G1, ATPase H^+^ transporting V1 subunit G1; TGF-β, transforming growth factor beta; CTGF, connective tissue growth factor (created with BioRender.com on June 2022).

Ectopic expression of stable HIF-1α in CAFs enhances aerobic glycolysis, lactate production, and autophagy. Chronic hypoxia activates HIF-1α/BNIP3-mediated autophagic degradation of Caveolin 1 (Cav-1), which reciprocally stabilizes HIF-1α via ROS production. Nutrients produced during glycolytic and autophagic metabolism are delivered to nearby cancer cells and promote cancer growth [88]. Moreover, TGF-β-mediated connective tissue growth factor (CTGF) overexpression alters HIF-1α-dependent cellular metabolism in fibroblasts, thereby supporting tumor growth [89] (Figure 3).

### 2.4. Angiogenesis

Vascular endothelial growth factor (VEGF) is one of the representative target genes of HIF that acts as a key factor in angiogenesis. VEGF can stimulate VEGFR on the surface of endothelial cells, then accelerate endothelial cell proliferation and angiogenesis [90]. In breast cancer, it has been shown that HIF-1α, in concert with G protein-coupled estrogen receptor 1 (GPER), transcriptionally upregulates VEGF expression in hypoxic CAFs to promote endothelial tube formation [91]. Generally, HIF-1α signaling is thought to promote cancer progression, but in some studies, HIF-1α was shown to be a negative regulator of cancer progression. HIF-mediated overexpression of angiogenic factors may promote abnormal angiogenesis, resulting in non-functional blood vessels and poor tumor perfusion. Fibroblast-specific HIF-1α, but not HIF-2α, knockout in mouse mammary tumor model resulted in the formation of normal vessel structure and enhanced tumor growth. Similar results were obtained when VEGF, a transcriptional target of HIF, was ablated. Deletion of VEGF in fibroblasts resulted in normal tumor vasculature, which in turn led to improved blood perfusion and tumor progression [92].

Stable isotope labeling by amino acids in cell culture (SILAC)-based quantitative proteomics analysis revealed that mammary CAFs secrete various angiogenic factors to promote blood vessel sprouting under hypoxia. Co-culture of patient-derived or cancer cell-transformed CAFs with HUVEC cells in hypoxia altered both intracellular and secretory proteins involved in cell metabolism and angiogenesis. It was found that a significant portion of the analyzed proteins did not belong to previously known HIF-1α target genes. Pro-angiogentic factor, stanniocalcin 1 (STC1) and anti-angiogentic factor, collagen type IV alpha 2 chain (COL4A2) were up- and down-regulated, respectively, by hypoxia-induced angiogenesis regulator (HIAR) in CAFs. Hypoxic CAFs also stimulated the secretion of VEGFA by expressing HIAR to promote the sprouting of endothelial cells [93]. Bevacizumab is an anti-angiogenic monoclonal antibody used for the treatment of various types of solid cancers. It neutralizes the VEGF ligand and inhibits angiogenesis. However, combination with other anti-cancer agents is required due to limited efficacy and/or adverse side effects [94]. As bevacizumab can reduce immune cell infiltration and suppress immune reactions, combination of bevacizumab and immune checkpoint blockade offers many clinical benefits in the treatment of cancer. Indeed, in phase I-III studies, the combinations of bevacizumab with PD-L1 inhibitors have shown synergistic effects and positive outcomes [95].

### 2.5. Metastasis

It has been shown that global haplodeficiency of PHD2 lowers tumor metastasis by inhibiting CAF activation. In a spontaneous polyomavirus middle T antigen (PyMT) mammary tumor model, PHD2 deletion suppressed ECM production and matrix contraction mediated by CAFs, which resulted in impaired cancer cell extravasation. In the PHD2-null mouse, paracrine interaction between cancer cells and CAFs was disrupted, which was mediated by TGF-β1 produced by cancer cells. Interestingly, decreased TGF-β1 secretion in cancer cells lacking PHD2 is likely independent of HIF as inhibition of HIF did not restore TGF-β1 production [96]. If that is the case, it is possible that PHD2 regulates CAF activation by targeting non-HIF substrates. Indeed, PHD and FIH enzymes have been found to hydroxylate several substrates other than HIF [97,98,99,100,101]. However, it should be noted that there has been a recent study that refutes the hydroxylation of some PHD substrates in vitro [102]. In another study, pharmacologic inhibition of HIF hydroxylases ameliorated intestinal fibrosis by suppressing TGF-β1-mediated activation of fibroblasts. This antifibrotic effect of hydroxylase inhibitors was independent of HIF and was at least partially due to inhibition of the extracellular regulated kinase (ERK) pathway [103].

Similarly, another study showed that chronic hypoxia suppresses the pro-tumorigenic remodeling of the tumor microenvironment by CAFs, thereby inhibiting tumor growth and metastasis. They have shown that both hypoxia and depletion of PHD2 in CAFs stabilize HIF-1α, which in turn reduce α-SMA and periostin expressions required for CAF-induced ECM remodeling and cancer cell invasion. In an orthotopic breast cancer model, inhibition of PHD2 by an HIF-hydroxylase inhibitor DMOG (dimethyloxalylglycine) reduces primary tumor stiffness and metastases of tumor cells to distant organs. Furthermore, co-injection of 4T1 breast cancer cells and PHD2-null CAFs prevents the CAF-induced metastasis of cancer cells to liver and lungs. Suppression of CAF-induced stromal remodeling and cell invasion by PHD depletion was dependent on HIF-1α as simultaneous depletion of HIF-1α prevented such events [104]. In support of these findings, HIF-1α knockout in cardiac fibroblasts was shown to increase tissue fibrosis following ischemic injury [105].

In pancreatic cancer, hypoxia upregulates HIF-1α expression in both cancer cells and fibroblasts. MRC5 fibroblast cells cultivated in hypoxia secrete hepatocyte growth factor (HGF) to increase c-Met phosphorylation and invasiveness of PK8 pancreatic cancer cells [106]. Hypoxic CAFs can induce EMT of cancer cells by altering the epigenetic transcriptional program. EMT enables distant metastasis by allowing epithelial cells to acquire mesenchymal properties such as reduced cell-cell contact and increased motility. In colorectal cancer model, hypoxia has been shown to induce CAF-mediated secretion of exosomes to promote cancer progression [107]. In PC3 prostate cancer cells, HIF-1α, NF-κB, and COX-2 pathways are activated by CAF-mediated ROS generation, leading to EMT and metastatic dissemination [108]. Curcumin (diferuloylmethane) has been shown to suppress CAF-mediated EMT and cell invasion by inhibiting MAOA/mTOR/HIF-1α-dependent oxidative response [70]. Reciprocally, by selectively removing hypoxic populations from tumors, it has been shown that hypoxic tumor cells affect CAF number and ECM composition. Genetically engineered PC3 cells expressing HRE-driven cytosine deaminase has been established to convert the nontoxic prodrug 5-fluorocytosine to active 5-fluorouracil under hypoxia. Significant reduction of CAFs and fiber volume were observed in PC3 xenograft model when hypoxic tumor cells were eliminated by 5-fluorouracil [109].

## 3. Targeting CAFs for Cancer Therapy

As CAFs play a major role in various cancer-promoting processes, inhibiting CAFs can be one of the effective strategies for cancer treatment. However, there is also evidence that CAFs inhibit cancer progression under certain circumstances, so it is necessary to consider which CAF subtypes should be targeted in which context [110]. Currently, a significant number of CAF-targeted cancer therapies are being developed, but most are in preclinical trials. Several different approaches have been proposed for CAF inhibition. Here, we will discuss potential anticancer agents targeting CAFs in the hypoxic tumor microenvironment (Table 1).

### 3.1. Targeting CAF Activation and Function

Efforts have been made to develop therapeutic agents that target the signaling pathways responsible for CAF activation or tumor-promoting factors secreted from the active CAFs. In the context of targeting CAFs in relation to the hypoxic tumor microenvironment, TGF-β, HIF, and CXCL12/CXCR4 signaling pathways are perhaps the most promising targets.

Currently, several TGF-β modulators are in phase I-III clinical trials for different cancers [17,130,131]. In preclinical studies, tranilast has been tested as an antifibrotic agent to inhibit CAF-mediated fibrosis by reducing TGF-β, CXCL12, and MMP2 in fibroblasts [111,112]. Similarly, in a rat type-2 diabetes model, tranilast has been shown to reduce the hypoxic induction of pro-inflammatory cytokines IL-1β, NF-κB, and TNF-α [113]. Pirfenidone, an anti-fibrotic agent for idiopathic pulmonary fibrosis, inhibits CAF activation and proliferation in lung and pancreatic cancers by targeting TGF-β and reduces the production of fibrotic mediators secreted by CAFs [114,115,116,117]. In hypoxic pulmonary hypertension, pirfenidone inhibits hypoxia-induced proliferation and migration of adventitial fibroblasts, thereby reducing the expression of α-SMA and collagen type I alpha 1 chain (COL1A1) [118]. Minnelide, a pro-drug of triptolide, downregulates TGF-β signaling in CAFs to enhance tumor regression in a mouse model of pancreatic cancer. It inhibits collagen stabilization and hyaluronan synthesis by reverting active CAFs into an inactive state [119,120]. In hypoxia, minnelide inhibits the transcriptional activity of HIF-1 by depleting its co-activator p300, thereby decreasing the stemness in pancreatic cancer [121]. Minnelide is currently in phase II trial against adenosquamous carcinoma of the pancreas (ASCP) [122].

Hypoxia induces TGF-β2 secretion from CAFs and activates HIF-1α to transcriptionally upregulate the expression of GLI family zinc finger 2 (GLI2) in colorectal cancer stem cells. GLI2 transcriptional factor has been implicated in carcinogenesis and chemoresistance in various solid malignancies. Co-culture of tumorsphere cells with CAFs under hypoxia promotes resistance to 5-fluorouracil (5-FU) and oxaliplatin treatment in a GLI2-dependent manner. Such hypoxia-induced chemoresistance was reversed by combination therapy with TGF-β inhibitor SD208 and GLI Inhibitor GANT61 [123]. TGF-β promotes MMP-mediated cleavage of CD44, a cell surface receptor for hyaluronic acid implicated in cancer cell invasion and metastasis [124]. CD44 is highly expressed in CAFs present in the avascular hypoxic regions of HT29 colorectal tumor mouse model. Moreover, co-culture of CD44-expressing CAFs and Lewis lung carcinoma (LLC) cells enhances the chemoresistance of LLC cells against 5-FU treatment by upregulating the expression of multidrug resistance protein 1 (MDR1) in cancer cells [132]. ERK1/2 inhibitor PD98059 and PI3K inhibitor LY294002 may be used to inhibit the TGF-β-mediated MMP/CD44 signaling by blocking the transduction pathway that mediates CD44 cleavage and activation [124].

HIF transcription factors continue to be of interest as therapeutic targets for cancer, and although some HIF inhibitors have shown considerable promise, their clinical applications are still limited. Developing selective HIF inhibitors remains a challenge. Direct HIF inhibitors may suppress mRNA expression, protein synthesis, alpha/beta dimerization, or transcriptional activity. Several drugs have been developed to indirectly inhibit HIF by modulating its upstream or downstream effector molecules [27,133,134,135,136]. Recently, FDA approved belzutifan, a small molecule inhibitor of HIF-2α, for the treatment of renal cell carcinoma patients associated with von Hippel–Lindau disease [137,138,139]. It should be noted that HIF in CAFs may either promote or inhibit cancer depending on the specific tumor context and microenvironment. CAF-specific HIF-depleting or -activating therapeutics should be developed and tested in preclinical models. In addition, HIF increases the expression of αvβ3 integrin at the surface of cancer cells, endothelial cells, and myofibroblasts, thereby promoting tumor cell motility [140,141]. ProAgio, a rationally designed protein agent, targets αvβ3 at a novel site and induces apoptosis of cells expressing high levels of αvβ3. In PDAC, where integrin αvβ3 is highly expressed, ProAgio targets cancer-associated pancreatic stellate cells (CAPaSC) to induce apoptosis and increase tumor permeability, leading to enhanced drug delivery [125,126]. ProAgio is currently in phase I clinical trial for pancreatic cancer.

CXCR4, a hypoxia-inducible chemokine receptor, interacts with CXCL12 to suppress CD8-positive cytotoxic T cells, thereby supporting immune evasion of tumor cells. CXCL12 is known to be produced mainly by fibroblast activation protein (FAP)-expressing CAFs in the tumor microenvironment [127]. In a mouse lung carcinoma model, depletion of FAP-expressing stromal cells causes acute cytokine-induced hypoxic death of both cancer and stromal cells [142]. In pancreatic ductal adenocarcinoma, combination therapy with anti-PD-L1 antibody and AMD3100, a selective CXCR4 antagonist, increases T cell accumulation in tumor tissue by suppressing CXCR4-mediated exclusion of cytotoxic T cells [127]. AMD3100 is an FDA-approved drug for patients with multiple myeloma or non-Hodgkin’s lymphoma who have undergone bone marrow transplantation [128]. Several other CXCR4 antagonists are being tested for cancer treatment in preclinical and clinical settings [129].

### 3.2. CAF Depletion

Various methods have been assessed to deplete CAFs residing in the tumor tissue. FAP is one of the highly expressed CAF markers in many epithelial cancers and is a potential target for CAF depletion. Genetic or pharmacological depletion of FAP-expressing CAFs reduces tumor growth in preclinical cancer models [143,144,145]. The aFAP-PE38 immunotoxin targeting FAP specifically depletes FAP-positive CAFs to inhibit angiogenesis and induce apoptosis, thereby reducing tumor growth. Combination of aFAP-PE38 with paclitaxel increased antitumor activity and prolonged survival in a mouse 4T1 breast cancer model [143]. Depletion of α-SMA-expressing CAFs promotes extensive remodeling of the tumor ECM and reduced tissue stiffness. In cholangiocarcinoma, α-SMA-positive CAF depletion by photothermal therapy (PTT) reduced tumor stiffness and growth. PTT is a physical therapy that induces hyperthermia by irradiating photoactivable nanoparticles with a near-infrared laser. Gold-decorated iron oxide nanoflower (GIONF)-mediated hyperthermia preferentially depleted CAFs in xenograft mouse model and contributed to tumor regression [146]. In another study, however, depletion of α-SMA-positive CAFs induced intratumoral hypoxia as it reduces secretion of pro-angiogenic factors and lowers vascular density. In a transgenic PDAC mouse model, genetic depletion of CAFs promoted tumor hypoxia, EMT, and cancer stemness. In addition, CAF depletion suppressed the infiltration of effector T cells associated with increased cytotoxic T-lymphocyte-associated protein 4 (CTLA-4) expression. Indeed, administration of CTLA-4 checkpoint blockade restored the overall survival of mice reduced by CAF depletion. Such results highlight the need for caution in establishing therapeutic strategies, as CAF depletion may rather promote cancer progression, depending on the circumstance [147].

## 4. Conclusions

Tumor hypoxia is a common feature of advanced cancers that can affect both cancer and stromal cells. Hypoxia-induced oncogenic signals modulate CAF phenotype and function to support cancer formation and progression. Moreover, cellular signatures appearing in hypoxia are induced not only by oxygen deprivation but also by mutations, ROS production, and metabolic changes associated with pseudohypoxia [148]. Hypoxic CAFs regulate ECM dynamics, immune response, cell metabolism, vessel formation, metastasis, and therapy resistance in cancer. Signaling pathways activated in hypoxic environments include TGF-β, HIF, and CXCL12/CXCR4 pathways. In general, targeting these molecules is expected to be an effective strategy for cancer treatment, but it should be noted that, depending on the role of hypoxic CAFs in certain cancers, in some cases, it may rather promote cancer progression. Several drugs that may affect cellular responses to hypoxia have been evaluated in preclinical and clinical studies. Some of these drugs have previously been approved by the FDA for other purposes or mechanisms, and it may be of interest to investigate their effects on hypoxic CAFs. In addition, it is necessary to check whether these drugs can be used in combination with currently used therapeutics to improve treatment efficacy.

## Figures and Tables

**Table 1:** Potential anticancer drugs targeting CAFs in the hypoxic tumor microenvironment.

Drugs	Mechanisms	Effects	Cancer Models	Status	References
Tranilast	TGF-β inhibition	Inhibits CAF-mediated fibrosis by reducing pro-inflammatory cytokines	Lymphoma, Lewis lung carcinoma, gastric cancer	Preclinical	[111,112,113]
Pirfenidone	TGF-β inhibition	Inhibits CAF activation and proliferation	Non-small-cell lung carcinoma, pancreatic cancer	Preclinical	[114,115,116,117,118]
Minnelide	TGF-β and HIF inhibition	Induces CAF inactivation	Pancreatic cancer	Phase I-II	[119,120,121,122]
SD208	TGF-β inhibition	Reduces CAF-induced chemoresistance	Colorectal cancer	Preclinical	[123]
GANT61	GLI inhibition	Reduces CAF-induced chemoresistance	Colorectal cancer	Preclinical	[123]
PD98059	ERK1/2 inhibition	Inhibits CAF signaling	Melanoma	Preclinical	[124]
LY294002	PI3K inhibition	Inhibits CAF signaling	Melanoma	Preclinical	[124]
ProAgio	αvβ3 inhibition	Induces apoptosis	Pancreatic cancer	Phase I	[125,126]
AMD3100	CXCR4 inhibition	Inhibits CAF-mediated immune evasion	Multiple myeloma, non-Hodgkin’s lymphoma, pancreatic ductal adenocarcinoma	Approved	[127,128,129]

TGF-β, transforming growth factor-β; GLI, GLI Family Zinc Finger; ERK1/2, extracellular regulated kinase 1/2; PI3K, phosphoinositide 3-kinase; CXCR4, C-X-C motif chemokine receptor 4.
